# FISH Analysis for del6q21 and del17p13 in B-cell Chronic Lymphocytic Leukemia in Iranians

**DOI:** 10.5812/ircmj.4990

**Published:** 2013-02-05

**Authors:** Hossein Teimori, Saeede Ashoori, Mohamad Taghi Akbari, Marjan Mojtabavi Naeini, Morteza Hashemzade Chaleshtori

**Affiliations:** 1 Cellular and Molecular Research Center, School of Medicine, Shahrekord University of Medical Sciences, Shahrekord, IR Iran; 2 Department of Medical Genetics, Faculty of Medical Sciences, Tarbiat Modares University, Tehran, IR Iran

**Keywords:** Leukemia, Lymphocytic, Chronic, B-Cell, Cytogenetic Aberrations, Biological Markers

## Abstract

**Background:**

B-cell chronic lymphocytic leukemia (B-CLL) is the most common leukemia in the Western world. Major progress has been made in assessing typical chromosomal abnormalities and recognition of the correlation of these chromosomal abnormalities with laboratory features and clinical course of the disease. The most frequent genomic changes are deletions at 13q14, 11q22-23 and 17p13 and trisomy of chromosome 12.

**Objectives:**

The aim of this study was to investigate the frequency of chromosomal aberrations in B-CLL patients’ peripheral blood and/or bone marrow using a molecular cytogenetic method, interphase fluorescence in situ hybridization (I-FISH) and to evaluate the correlation between these genomic changes and clinical findings.

**Patients and Methods:**

I-FISH analyses were performed on bone marrow and blood samples of 66 B-CLL patients.

**Results:**

Deletion of 17p13 was found in 11 (16.6%) and deletion 6q21 was present in 5 (7.5%). Statistical analyses were performed to investigate the correlation of these molecular-cytogenetic findings with family history, Rai staging and CD38 marker. No clear differences in distribution was noted for del17p13 and del6q21 among patients with and without family history, and no direct correlation was noted between these genomic changes and CD38 marker, but the correlation of del17p13 and Rai stage was significant. There was a high frequency of Rai stage II within del17p13 patients.

**Conclusions:**

It was demonstrated that the presence of del6q21 in B-CLL patients indicates poor prognosis and on the contrary, presence of del17p13 points at the good prognostic value of the disease.

## 1. Background

B-cell chronic lymphocytic leukemia (B-CLL) is usually described as the most common leukemia in the United States, Canada, and Western Europe, whereas it is rare in Japan and infrequent in other Asian societies (1). CLL is with an incidence of 30% among the entire leukemia’s (2) as the matter of fact it is 20 new cases per 100,000 inhabitants above the age of 60 years. The reported male-to-female sex ratio is about 1.5–2:1, which means B-CLL is more common in men than women (3). It is a common cancer not even in the Western countries, but also in Iran (4). B-CLL results from the expansion of mature-appearing monoclonal B cells with a characteristic immunophenotype (CD5, CD19, CD20, and CD23 positive) (5-7). Clinical course of the disease is highly variable. Some patients show symptoms at diagnosis or early thereafter and need early therapy, but others have no or minimal symptoms for many years (8). Infrequently, there are constitutional symptoms; on the contrary, the most frequent physical finding is lymphadenopathy, which is followed by splenomegaly. Other symptoms are fatigue, infection and autoimmune hemolytic anemia. There are several treatments for B-CLL patients such as using monoclonal antibodies, new experimental drugs and stem cell transplantation (autologous or allogeneic) (9). Variable prognosis is carried by B-CLL, which is associated with distinct parameters such as genetic aberrations that are helpful to predict the clinical outcome of the disease (10, 11). Age, sex, and clinical staging (Rai or Binet staging), peripheral blood lymphocyte count, lymphocyte-doubling time, bone marrow (BM) histology, serum thymidine kinase, and serum β2-microglobulin levels have all been identified as important independent prognostic factors (12-15). Chromosomal aberrations in B-CLL rose to more than 80% (16-18), which means that genetic markers could have a critical role in prediction and diagnosis of B-CLL. The most frequent aberrations are: 1) Deletion on the long arm of chromosome 13 (13q14) in > 50% of the cases (19); 2) Trisomy of chromosome 12 in 10–20% of the cases; 3) Deletions in bands 11q22–q23 in 10–20% of the cases, where the ATM gene is located (20-22); 4) Deletion on the short arm of the chromosome 17 (17p13). Association of Deletion 11q22 and 17p13 with poor prognosis and deletion 13q14 with good prognosis has been demonstrated (23). Trisomy 12 is correlated with atypical morphology and shortened survival (worse prognosis) (24, 25). Conventional cytogenetic methods can detect 40-50% abnormalities in B-CLL patients (26, 27), while using fluorescent in situ hybridization (FISH) has brought the sensitivity of cytogenetic analysis to a higher level, by which, 80% of abnormalities can be detected (28). In the previous study, we analyzed the correlation of del (13q), del (11q) and trisomy 12 with features of B-CLL in Iranian cases (29).

## 2. Objectives

The aims of this study were first to verify the frequency of del (6q21) and del (17p13) in B-CLL patients by conventional cytogenetic methods and FISH technique, and second to determine the correlation between these two abnormalities and prognostic factors, including Rai staging, CD38 marker and family history.

## 3. Patients and Methods

Patients were recruited for 20 months between 2008 and 2010 from four major hematology/oncology hospitals in Tehran, Iran. The patients were new cases or already identified as B-CLL cases and not taking any therapeutic treatment for six months before sampling. The diagnostic inclusion criteria were based on the National Cancer Institute Working Group (NCI-WG) guidelines for diagnosis of B-CLL (30). Seventy patients’ peripheral blood samples or bone marrow aspirations were analyzed. Immunophenotypic data and blood parameters were collected from patients’ hospital files. The questions about age, family history, the onset of disease and treatment measures were organized. The patients with one first or two second degree relatives affected by any type of cancer in their families were considered as positive family history. Blood samples and/or bone marrow aspirations were collected in heparinized collection tubes. The study was approved in Shahrekord Medical University and Ethical Committee of the School of Medical Sciences of Tarbiat Modares University. Each patient signed the written consent. Peripheral blood and bone marrow samples were pretreated by different protocols for culturing. 27 bone marrows, 36 blood samples and 7 of both specimens were provided. Peripheral blood was washed three times with minimal culture medium (RPMI-1960 with no further supplementation) and then the white blood cells (WBC) were counted by Neubauerhemocytometer. Bone marrow specimens only were counted by Neubauerhemocytometer. 106 cells/ml was cultured in 5 ml completed culture medium [RPMI-1960 medium (Gibco, USA), 10% fetal bovine serum (Gibco, USA), 1% antibiotics and 1% L-glutamine (Gibco, USA)]. Five cultures were set up for each patient whose conditions were as follows: overnight (ONC), 24 hours and 72 hours without mitogen stimulation and 24 hours and 72 hours with phorbolmyristate acetate (50 ng/mL) (Biomol, Germany). Harvesting and slide preparations were completed according to the standard cytogenetic methods (Hypotonic treatment, and methanol-acetic acid, Merk, Germany; 3:1 ratio of fixation). G-banding technique was used and up to 20 metaphases were analyzed for each patient utilizing Applied Imaging Powergene Intelligent Karyotyping software (Applied Imaging, USA) according to the International system for Human Cytogenetic Nomenclature (31). The FISH analysis was performed on slides taken from the 24-hour culture without stimulation. To evaluate del (6q21) and del (17p13), Dual-color probes were purchased from Kreatech Company and utilized according to the manufacturer’s instructions. For each probe per patient, two hundred nuclei were analyzed by Olympus BX51 fluorescence microscope (Japan). To capture images, Cytovision software, version 3.6, was used. SPSS software program (SPSS for Windows, version 15, Chicago, IL, USA) was used for statistical analysis. Correlations between family history and these two deletions were analyzed by Chi-Square test. The Mann-Whitney test was considered for analyzing the correlation between the Rai staging and chromosomal abnormalities. The Mann-Whitney test was used to evaluate the correlation between these chromosomal abnormalities and CD38. A p value less than 0.05 were considered significant.

## 4. Results

The culture of four cases failed, and 66 specimens were successfully analyzed. In Table 1, patients’ clinical characteristics are summarized. The gender ratio was 2.9 with 49 male and 17 female patients. Patients were 40 to 81 years old with the mean age of 61.73 years old. They could be classified into two subgroups. Twenty three cases have been recently diagnosed (new cases), and they were following their clinical diagnosis. Forty three cases suffered from the disease from 8 to 236 months. The number of bone marrow specimens in Table 1 is actually the sum of cases with bone marrow and cases with both bone marrow and peripheral blood samples. The information of Rai staging and family history is also available in Table 1.

**Table 1.** Clinical and Laboratory Features of Iranian B-Cell Patients

Patients	Genomic Changes		Total (n = 66)
	Del 6q21 (n = 4)	Del 17p13 (n = 11)	
**Gender**, No.			
Male	1	10	49
Female	3	1	17
**Age, y, No.**			
< 50	0	1	8
50-60	0	4	17
60-70	2	4	25
> 70	2	2	16
**Tissue**			
BM a	2	6	27
PB a	3	6	32
**Rai stage**			
O	0	0	6
I	0	1	9
II	3	3	31
III	1	6	15
IV	0	1	5
**Family history**			
Positive	1	9	15
Negative	3	2	47

^a^Abbreviation: BM, bone marrow; PB, peripheral blood

Interphase FISH for del (6q21) and del (17p13) was performed on 66 patients; out of which 5 cases demonstrated the micro deletion in 6q21 (5/66 or 7.5%); and 11 cases turned out to have the micro deletion in 17p13 (11/66 or 16.6%); among these 16 patients with these chromosomal abnormalities, one patient had both del (6q21) and del (17p13). The range of abnormalities was different in patient’s specimens. Compared to cut off points for positive values, this range verified the abnormalities in patients properly. Correlation of family history and del (17p13) were analyzed by Chi square (P = 0.48 for Fisher’s exact test) and did not show any significance. This test also was performed between family history and del (6q21) (P = 1.000 for Fisher’s exact test) but the correlation was not significant. The Mann-Whitney test was considered for analyzing the correlation between Rai staging and del (6q21). The result was P = 0.7, which means there is no significance. This test was repeated for the correlation of chromosomal abnormalities and CD38. The result for del (6q21) and del (17p13) was P = 0.368 and P = 0.13 respectively, which means CD38 did not have significance in any of these two abnormalities. FISH analysis revealed 22.7% chromosomal abnormalities in total comprising 7.5% for del (6q21) and 16.6% for del (17p13). The results of other published studies and the current study are summarized in Table 2.

**Table 2.** Detection Rate of Cytogenetic Aberrations byFIsh Analysis in Different Populations

No	Reference	Country	Del 17p13, %	Del 6q21, %	FISH Abnormality, No. (%)
**1**	Gunn et al. (32)	USA	4.6	7.5	174 (89)
**2**	Gaidano et al. (33)	USA	4	10	100 (14)
**3**	Dohner et al. (34)	Germany	12	-	90 (12)
**4**	Dohner et al. (17)	Germany	7	6	325(82)
**5**	Chevallier et al. (35)	France	7	-	111 (75)
**6**	Haferlach et al. (36)	Germany	**7**	4.6	500 (78.4)
**7**	Juliusson et al. (37)	Sweden	4	6	649 (48)
**8**	Turgut et al. (38)	Turkey	14	-	36 (47)
**9**	Sindelarova et al. (39)	Czech republic	16	-	206 (16)
**10**	Durak et al. (40)	Turkey	7.6	-	79 (50.6)
**11**	Dewald et al. (41)	USA	8	0	113 (77)
**12**	Dicker et al. (42)	USA	5.3	7	132 (79)
**13**	Present study	Iran	16.6	7.5	66 (22.7)

## 5. Discussion

CLL is the most prevalent type of leukemia, but its annual incidence of new cases is low and consequently; the number of new cases is generally low (4). So in the present study the number of new cases hardly comprises one-third of all cases. The gender ratio shifted towards more males because, some female patients avoided to take part in the study. There were two reasons why bone marrow specimens were less than peripheral blood samples. First, the greater numbers of cases were those who had already been diagnosed as B-CLL and the physicians did not feel necessary to perform another bone marrow aspiration; second, an analysis of both bone marrow and blood samples demonstrated that there are no differences between these two tissues in FISH results and patients prefer to give blood samples instead of bone marrow aspiration. So the number of peripheral blood specimens increased in this study. The weak mitogen stimulation of malignant cells in B-CLL and the fact that in B-CLL unlike other forms of leukemia, the cells are in G0 phase of the cell cycle, we had a decrease of metaphases in the cytogenetic analysis. Moreover, many of the chromosomes were small and short and less liable to good banding. So it was so difficult to identify chromosomal abnormalities such as translocations, inversions and micro deletions. Because of these reasons, we could just identify 27.7% chromosomal abnormalities using conventional cytogenetic methods.

As mentioned before, up to 80% of chromosomal abnormalities in B-CLL patients could be detected using I-FISH (43). Oscier et al. (44), using FISH; found chromosomal aberrations in 69% of patients , Dewald et al. (41), found 77%; Chena et al. (2), 80.7%; Gunn et al. (32), 89%; and Dohner et al. (17), 82%. In some of these studies (17, 41), B-CLL patients were examined for deletion of 13q, 11q, and 17p; also for 6q, 14q chromosomal abnormalities and for 8q24 and 3q trisomy (17). So the rate of chromosomal aberrations detected by FISH is high. The greater detection of chromosomal abnormalities by FISH method in comparison to conventional cytogenetic methods reveals the higher sensitivity of this method. Therefore, FISH testing helps outstandingly in establishing a diagnosis for these patients. In our study where I-FISH was only used for two genomic changes and patients were examined with two DNA probes (for detection of deletion 6q21 and deletion 17p) we could identify these abnormalities in just 22.7% of patients, comprising 16.6% for del 17p13 and 7.5% for del 6q21. The results of this study and some similar studies are summarized in Table 2. We found deletion of TP53 gene on 17p13 in 11 (16.6%) of studied cases. In addition to deletion of 17p13 chromosomal band or occurring mutations in TP53 gene, inactivation of this gene could be because of other factors. For example Pettitt et al. (45) revealed that p53 inactivation occurred because of defects of ATM protein, which has a role in dephosphorylation of p53. Some other studies have shown that deletion of 17p13 band has a significant effect on clinical course of the disease. B-CLL patients with deletion of TP53 gene have an obvious shorter survival time and show failure to treatment with purine analogs (17, 34, 46, 47). Anomalies of short arm of chromosome 6 are reported from 0-10% (33, 36, 41). In this region most abnormalities are deletions and diverse regions have been identified at 6q15, 6q21, 6q23 and 6q25-27 (48, 49). Stilgenbauer et al. showed that 6q21 is the most commonly deleted region and 6q27 deletion occurs only in patients with del (6q21) (50). The genes in this region which are involved in the pathogenesis of the disease are not known yet but the TLX gene at 6q21 has been shown to be involved in non-Hodgkin’s lymphoma patients (51, 52). In analysis of clinical importance of 6q deletions in B-CLL patients contentious results were observed. While Juliusson et al. (37) didn’t observe any poor prognostic effect of del (6q), Oscier et al. (53) observed that those patients with del (6q) had shorter survival times without treatment. In our study, del (6q21) was detected in 5 (7.5%) of studied cases. One of the patients had both abnormalities, del (6q21) and del (17p13). As it is evident, the frequency of patients with del (17p13) and del (6q21) and so the type and the rate of these abnormalities in Iranian patients is similar to those in other populations. This suggests that probable mechanisms involved in this disease are not different from patients from other countries.

As far as, correlation between clinical staging, family history and immunophenotyping with these two cytogenetic subgroups is concerned, after statistical analysis, significant correlation was only shown between Rai staging and del (17p13). This deletion has the most frequency in Rai stage II, so Rai stage II could be somehow a prognostic factor for this deletion. We can arrange Rai stages in descending sort (II, III, I, O, IV) for this correlation. Cases with familial B-CLL comprise about 5% of B-CLL patients. Cytogenetic and immunophenotypic results of familial and sporadic B-CLL patients were similar to each other. Some other studies analyzing familial B-CLL cases have also suggested similar findings (54, 55).

## References

[1] Faguet GB (2004). Chronic Lymhocytic Leukemia: Molecular Genetics, Biology, Diagnosis, and Management..

[2] Chena Christian, Arrossagaray Guillermo, Scolnik Mariano, Palacios María F, Slavutsky Irma (2003). Interphase cytogenetic analysis in Argentinean B-cell chronic lymphocytic leukemia patients: association of trisomy 12 and del(13q14).. Cancer genetics and cytogenetics..

[3] Rozman C, Montserrat E (1995). Chronic lymphocytic leukemia.. N Engl J Med..

[4] Karimi M, Mehrabani D, Yarmohammadi H, Jahromi FS (2008). The prevalence of signs and symptoms of childhood leukemia and lymphoma in Fars Province, Southern Iran.. Cancer Detect Prev..

[5] Caligaris-Cappio F, Hamblin TJ (1999). B-cell chronic lymphocytic leukemia: a bird of a different feather.. J Clin Oncol..

[6] Dierlamm J, Michaux L, Criel A, Wlodarska I, Van den Berghe H, Hossfeld DK (1997). Genetic abnormalities in chronic lymphocytic leukemia and their clinical and prognostic implications.. Cancer Genet Cytogenet..

[7] Hamblin TJ, Davis Z, Gardiner A, Oscier DG, Stevenson FK (1999). Unmutated Ig V(H) genes are associated with a more aggressive form of chronic lymphocytic leukemia.. Blood..

[8] Zenz T, Dohner H, Stilgenbauer S (2007). Genetics and risk-stratified approach to therapy in chronic lymphocytic leukemia.. Best Pract Res Clin Haematol..

[9] Pangalis GA, Vassilakopoulos TP, Dimopoulou MN, Siakantaris MP, Kontopidou FN, Angelopoulou MK (2002). B-chronic lymphocytic leukemia: practical aspects.. Hematol Oncol..

[10] Amiel A, Leopold L, Gronich N, Yukla M, Fejgin MD, Lishner M (2006). The influence of different chromosomal aberrations on molecular cytogenetic parameters in chronic lymphocytic leukemia.. Cancer Genet Cytogenet..

[11] Oscier D (2005). Biology and prognostic factors in CLL.. Hematology..

[12] Binet JL, Auquier A, Dighiero G, Chastang C, Piguet H, Goasguen J (1981). A new prognostic classification of chronic lymphocytic leukemia derived from a multivariate survival analysis.. Cancer..

[13] Doneda Luisa, Montillo Marco, Intropido Liliana, Tedeschi Alessandra, Morra Enrica, Larizza Lidia (2003). Interphase fluorescence in situ hybridization analysis of del(11)(q23) and del(17)(p13) in chronic lymphocytic leukemia: a study of 40 early-onset patients.. Cancer genetics and cytogenetics..

[14] Rai KR, Sawitsky A, Cronkite EP, Chanana AD, Levy RN, Pasternack BS (1975). Clinical staging of chronic lymphocytic leukemia.. Blood..

[15] Shanafelt TD, Geyer SM, Kay NE (2004). Prognosis at diagnosis: integrating molecular biologic insights into clinical practice for patients with CLL.. Blood..

[16] Bentz M, Huck K, du Manoir S, Joos S, Werner CA, Fischer K (1995). Comparative genomic hybridization in chronic B-cell leukemias shows a high incidence of chromosomal gains and losses.. Blood..

[17] Dohner H, Stilgenbauer S, Benner A, Leupolt E, Krober A, Bullinger L (2000). Genomic aberrations and survival in chronic lymphocytic leukemia.. N Engl J Med..

[18] Odero MD, Soto JL, Matutes E, Martin-Subero JI, Zudaire I, Rao PH (2001). Comparative genomic hybridization and amplotyping by arbitrarily primed PCR in stage A B-CLL.. Cancer Genet Cytogenet..

[19] Calin GA, Dumitru CD, Shimizu M, Bichi R, Zupo S, Noch E (2002). Frequent deletions and down-regulation of micro- RNA genes miR15 and miR16 at 13q14 in chronic lymphocytic leukemia.. Proc Natl Acad Sci U S A..

[20] Bullrich F, Rasio D, Kitada S, Starostik P, Kipps T, Keating M (1999). ATM mutations in B-cell chronic lymphocytic leukemia.. Cancer Res..

[21] Schaffner C, Stilgenbauer S, Rappold GA, Dohner H, Lichter P (1999). Somatic ATM mutations indicate a pathogenic role of ATM in B-cell chronic lymphocytic leukemia.. Blood..

[22] Stankovic T, Weber P, Stewart G, Bedenham T, Murray J, Byrd PJ (1999). Inactivation of ataxia telangiectasia mutated gene in B-cell chronic lymphocytic leukaemia.. Lancet..

[23] Sanchez J, Aventin A (2007). Detection of chromosomal abnormalities in chronic lymphocytic leukemia increased by interphase fluorescence in situ hybridization in tetradecanoylphorbol acetate-stimulated peripheral blood cells.. Cancer Genet Cytogenet..

[24] Criel A, Verhoef G, Vlietinck R, Mecucci C, Billiet J, Michaux L (1997). Further characterization of morphologically defined typical and atypical CLL: a clinical, immunophenotypic, cytogenetic and prognostic study on 390 cases.. Br J Haematol..

[25] Hjalmar V, Kimby E, Matutes E, Sundstrom C, Jacobsson B, Arvidsson I (1998). Trisomy 12 and lymphoplasmacytoid lymphocytes in chronic leukemic B-cell disorders.. Haematologica..

[26] Gahrton G, Robert KH, Friberg K, Zech L, Bird AG (1980). Nonrandom chromosomal aberrations in chronic lymphocytic leukemia revealed by polyclonal B-cell-mitogen stimulation.. Blood..

[27] Hurley JN, Fu SM, Kunkel HG, Chaganti RS, German J (1980). Chromosome abnormalities of leukaemic B lymphocytes in chronic lymphocytic leukaemia.. Nature..

[28] Montillo M, Hamblin T, Hallek M, Montserrat E, Morra E (2005). Chronic lymphocytic leukemia: novel prognostic factors and their relevance for risk-adapted therapeutic strategies.. Haematologica..

[29] Teimori H, Akbari M, Toogeh G, Khaleghian M (2010). Correlation of del13q, del11q and Trisomy 12 with Laboratory and Clinical Features of Chronic Lymphocytic Leukemia in Iranian Patients.. Iran Red Crescent Med J..

[30] Cheson BD, Bennett JM, Grever M, Kay N, Keating MJ, O'Brien S (1996). National Cancer Institute-sponsored Working Group guidelines for chronic lymphocytic leukemia: revised guidelines for diagnosis and treatment.. Blood..

[31] Mitelman F (1995). International System for Human Cytogenetic Nomenclature ISCN Table: The Normal Human Karyotype G- and R-Bands..

[32] Gunn SR, Mohammed MS, Gorre ME, Cotter PD, Kim J, Bahler DW (2008). Whole-genome scanning by array comparative genomic hybridization as a clinical tool for risk assessment in chronic lymphocytic leukemia.. J Mol Diagn..

[33] Gaidano G, Newcomb EW, Gong JZ, Tassi V, Neri A, Cortelezzi A (1994). Analysis of alterations of oncogenes and tumor suppressor genes in chronic lymphocytic leukemia.. Am J Pathol..

[34] Dohner H, Fischer K, Bentz M, Hansen K, Benner A, Cabot G (1995). p53 gene deletion predicts for poor survival and non-response to therapy with purine analogs in chronic B-cell leukemias.. Blood..

[35] Chevallier P, Penther D, Avet-Loiseau H, Robillard N, Ifrah N, Mahe B (2002). CD38 expression and secondary 17p deletion are important prognostic factors in chronic lymphocytic leukaemia.. Br J Haematol..

[36] Haferlach C, Dicker F, Schnittger S, Kern W, Haferlach T (2007). Comprehensive genetic characterization of CLL: a study on 506 cases analysed with chromosome banding analysis, interphase FISH, IgV(H) status and immunophenotyping.. Leukemia..

[37] Juliusson G, Oscier D, Gahrton G, Fitchett M, Ross FM, Brito-Babapulle V (1991). Cytogenetic findings and survival in B-cell chronic lymphocytic leukemia. Second IWCCLL compilation of data on 662 patients.. Leukemia & Lymphoma..

[38] Turgut B, Vural O, Pala FS, Pamuk GE, Tabakcioglu K, Demir M (2007). 17p Deletion is associated with resistance of B-cell chronic lymphocytic leukemia cells to in vitro fludarabine-induced apoptosis.. Leuk Lymphoma..

[39] Sindelarova L, Michalova K, Zemanova Z, Ransdorfova S, Brezinova J, Pekova S (2005). Incidence of chromosomal anomalies detected with FISH and their clinical correlations in B-chronic lymphocytic leukemia.. Cancer Genet Cytogenet..

[40] Durak B, Akay OM, Aslan V, Ozdemir M, Sahin F, Artan S (2009). Prognostic impact of chromosome alterations detected by FISH in Turkish patients with B-cell chronic lymphocytic leukemia.. Cancer Genet Cytogenet..

[41] Dewald GW, Brockman SR, Paternoster SF, Bone ND, O'Fallon JR, Allmer C (2003). Chromosome anomalies detected by interphase fluorescence in situ hybridization: correlation with significant biological features of B-cell chronic lymphocytic leukaemia.. Br J Haematol..

[42] Dicker F, Schnittger S, Haferlach T, Kern W, Schoch C (2006). Immunostimulatory oligonucleotide-induced metaphase cytogenetics detect chromosomal aberrations in 80% of CLL patients: A study of 132 CLL cases with correlation to FISH, IgVH status, and CD38 expression.. Blood..

[43] Stilgenbauer S, Lichter P, Dohner H (2000). Genetic features of B-cell chronic lymphocytic leukemia.. Rev Clin Exp Hematol..

[44] Oscier DG, Gardiner AC, Mould SJ, Glide S, Davis ZA, Ibbotson RE (2002). Multivariate analysis of prognostic factors in CLL: clinical stage, IGVH gene mutational status, and loss or mutation of the p53 gene are independent prognostic factors.. Blood..

[45] Pettitt AR, Sherrington PD, Stewart G, Cawley JC, Taylor AM, Stankovic T (2001). p53 dysfunction in B-cell chronic lymphocytic leukemia: inactivation of ATM as an alternative to TP53 mutation.. Blood..

[46] Dohner H, Stilgenbauer S, Dohner K, Bentz M, Lichter P (1999). Chromosome aberrations in B-cell chronic lymphocytic leukemia: reassessment based on molecular cytogenetic analysis.. J Mol Med (Berl)..

[47] Stilgenbauer S, Bullinger L, Lichter P, Dohner H (2002). Genetics of chronic lymphocytic leukemia: genomic aberrations and V(H) gene mutation status in pathogenesis and clinical course.. Leukemia..

[48] Juliusson G, Oscier DG, Fitchett M, Ross FM, Stockdill G, Mackie MJ (1990). Prognostic subgroups in B-cell chronic lymphocytic leukemia defined by specific chromosomal abnormalities.. N Engl J Med..

[49] Offit K, Louie DC, Parsa NZ, Filippa D, Gangi M, Siebert R (1994). Clinical and morphologic features of B-cell small lymphocytic lymphoma with del(6)(q21q23).. Blood..

[50] Stilgenbauer S, Bullinger L, Benner A, Wildenberger K, Bentz M, Dohner K (1999). Incidence and clinical significance of 6q deletions in B cell chronic lymphocytic leukemia.. Leukemia..

[51] Jackson A, Panayiotidis P, Foroni L (1998). The human homologue of the Drosophila tailless gene (TLX): characterization and mapping to a region of common deletion in human lymphoid leukemia on chromosome 6q21.. Genomics..

[52] Sherratt T, Morelli C, Boyle JM, Harrison CJ (1997). Analysis of chromosome 6 deletions in lymphoid malignancies provides evidence for a region of minimal deletion within a 2-megabase segment of 6q21.. Chromosome Res..

[53] Oscier DG, Stevens J, Hamblin TJ, Pickering RM, Lambert R, Fitchett M (1990). Correlation of chromosome abnormalities with laboratory features and clinical course in B-cell chronic lymphocytic leukaemia.. Br J Haematol..

[54] Aoun P, Zhou G, Chan WC, Page C, Neth K, Pickering D (2007). Familial B-cell chronic lymphocytic leukemia: analysis of cytogenetic abnormalities, immunophenotypic profiles, and immunoglobulin heavy chain gene usage.. Am J Clin Pathol..

[55] Ng D, Toure O, Wei MH, Arthur DC, Abbasi F, Fontaine L (2007). Identification of a novel chromosome region, 13q21.33-q22.2, for susceptibility genes in familial chronic lymphocytic leukemia.. Blood..

